# Case in Practice

**Published:** 1878-02-20

**Authors:** T. Brockway

**Affiliations:** Pine Meadow, Conn.


### CASE IN PRACTICE.

By T. Brockway, M.D., Pine Meadow, Conn.

I was called, on the morning of October 20, 1877, to visit the daughter of Mr. James Forbes, of this place. The child had swallowed a small copper cent two days previous to my being called. The coin had been lost some three weeks, and lain upon the ground that length of time before the child swallowed it. The usual remedy (oil) was given the child by its parents; evacuations followed, but the coin was not found. The mother gave more oil on the next day, and free evacuations followed, but the coin was still missing. At night, the child retired with its father, and was restless through the night; passed its urine involuntarily, which was something new for the child to do.

On the following morning, the child was bright, and feeling as well as usual. At the breakfast table, the first complaint came from the child, which was of a pain in its stomach. The family thought it the result of the oil, and asked the child if it wished the vessel, but it declined. A few moments more elapsed, and it again complained, and at this time did have an evacuation from the bowels, but the coin was not found.

The morning meal being through, the father went to his employment; the child retired from the table, and was looking from the window, when it called to its mother and exclaimed, "I am cross-eyed," and the next moment could not see. It was at this stage of the case that I was called. I hastened to the house, and found the child in the following condition: it lay upon its back, its eyes open and fixed; respiration had ceased; the tongue protruded over the teeth, and was swollen; the lips and tongue were of a deep blue color, and the body generally looking blue; the fingers were clenched in the hand, not with the thumb in the palm, but like a cramp. The child drew its chin on one side twice after I reached the house, not gasping, but simply a movement of the lower jaw. Before I reached the house, the child's clothes were loosened, and friction used over the stomach and abdomen, but the child could not bear it, and, as the lady expressed it, she acted like a worm on a hot stove. I could find no pulse when I reached the house; nothing but the fingers clenched indicated any spasmodic action. The extremities were cold and thoroughly relaxed, with the exception of the fingers. No discoloration under finger-nails.

The family, thinking the coin was still retained in the child, desired a *post-mortem* examination, which I performed the next day. I first dissected the stomach, which was found in the following condition: it was partially filled with a liquid, looking like milk and water. The lining membrane was contracted, and looked of a deep bronze color. The discoloration was deeper as it reached the pylorus. No inflammatory action about the stomach, nor the pyloric orifice. Nothing inflammatory about the duodenum. The small intestines showed inflammation and putrefaction through their whole extent, which, in my opinion, was caused by the passage of the coin. The coin was not found by the examination, but found in the privy vault. It was brought to me with the *debris* still upon it from the privy. I cleansed it, and found portions of it, as though it had been in acid. I concluded the coin was deeply corroded before the child swallowed it.

I find the homœopathic provings of cuprum give this child's symptoms exactly, and I could not but pronounce the case one of copper poisoning. The length of time from the first complaint at the table until the death of the child was scarcely three-fourths of an hour.

Now, as this case is the first one of the kind I have ever had, or heard of, I report it for publication, and hope for information in regard to it—especially the cause of no pain, and still the amount of inflammation. Did the copper produce paralysis of the bowels?—*Ec. Med. Journal.*

